# Peptonised Iodine in Tuberculosis

**Published:** 1911-06-24

**Authors:** L. Dieupart, S. Bernheim, A. Baud

**Affiliations:** Budapest; Paris; Paris; Assistant de l'Euvre de la Tuberculose Humaine. Paris


### Editor's Letter-Box.

### PEPTONISED IODINE IN TUBERCULOSIS.

A Reply to Our Criticism.

*To the Editor of* The Hospital.

Œuvre de la Tuberculose Humaine
(Siège Social : 9 Rue de Bellefond, Paris).

Sir,—It is only to-day that your copy of June 3, wherein you speak of the new treatment of tuberculosis by radioactive menthol iodine, has been communicated to us. Although we decided never to accept polemical controversy regarding our recent communication to the scientific societies, we cannot, without protest, pass by the suggestions and allegations published in your review. By not answering these altogether erroneous imputations we fear, indeed, that a certain number of colleagues in your country would be misled and give a wrong interpretation to our merely scientific work.

In The Hospital of June 3 you mention that the results of our experiments are vague and exiguous; that in our communication we do not state the number of experiments on animals; that our clinical observations themselves are totally lacking in thoroughness and precision; that, in a word, we have published a premature work and caused hopes to be raised in the minds of the public, and especially of sufferers from tuberculosis—hopes that will prove to be for them so many disappointments and disenchantments. You add that the chemical composition of radioactive menthol iodine is incompletely determined, and at least that we were wrong in making our work known through the daily lay Press instead of confiding firstly our results to the scientific societies and reviews. For these reasons, you say, you advocate complete scepticism, and advise our colleagues to show the greatest reserve.

We beg leave to answer each of these allegations, which are perfectly inexact. We are all the more surprised to hear them from you, as you have known us for a very long time by our personal works and our frequent discussions with Robert Koch at the time of his discovery of the tuberculine, and more of late with Professor von Behring with reference to his T.X. (Tulase). You know, consequently, that we are no novices in the study and researches regarding tuberculosis.

We are also surprised at your suggestions, as we sent you a note, with request to publish same in your paper, giving a detailed summary of our communication to the International Society of Tuberculosis, long before any daily paper had spoken of our researches.

This communication was made to forty-two French and foreign scientific societies, which came first to know our work, within an interval going from May 10 to 24 last. You know as well as we do that most of these scientific societies are open to the public and the Press, and that the latter is always excessively in a hurry to publish an original and curious work long before the professional reviews. It is the same with all the scientific works that are communicated to academies and scientific societies, and you cannot accuse the author of these works of having been the instigator of such publicity.

You affirm that our work is premature. Is this reproach a serious one? You have read, however, in the note we sent you that the researches commenced three years ago, and that one of our collaborators, Dr. de Szendeffy, made a year ago a previous communication on the results of our experiments on animals. These experiments are as little vague as our clinical observations themselves, as Mr. de Szendeffy repeated them more than forty times with animal controls, and all the experiments made in the same conditions succeeded each other and arrived at the same result.

In our investigations mention is only made of seventy-five clinical observations, although we submitted to the same treatment more than 150 patients, because we wished only to publish observations on sick ones kept under the most rigorous and most scientific observation.

In order to be protected from criticism and scepticism, we had the radioactive menthol iodine examined in different hospitals by physicians who did not know each other, and we took care to have the bacteriological examinations and chemical analyses made in laboratories not depending on our enterprise, and especially by the Municipal Laboratory of the City of Paris, under the direction of Dr. Miquel. You see, Sir, we carried our scrupulousness very far, and we proceeded in our researches with extreme probity.

Since writing our memoir we confided the radioactive menthol iodine to a very great number of hospitals and dispensaries, and already several of the best-known clinical physicians have informed us of results identical to ours. We are assembling all these new documents, which will be the object of a subsequent communication to the next International Congress of Tuberculosis in Rome.

When carefully reading our work in the original text nobody would doubt that our results are scientific and truthful. They raise in no way hopes that will be followed by disappointment, for we had the courage to give the indications and counter-indications of this new medicine, to indicate the cases where success may be expected, and, on the other hand, to mention the conditions wherein no hope is left.

What remains of your imputations? Premature work? Have you often seen learned men make with success serious researches who had the courage to keep silent for three years? Communications to the daily and lay papers. We never solicited the assistance of any political paper or took the responsibility of any article that was not published by ourselves. On the contrary, we sent communications to all French and foreign medical papers. A great number of them published already a summary of our work; many of them, and not the least ones, asked us permission to publish it in extenso.

A last point to terminate this justified answer. You speak somewhat inconsiderately of the chemical composition of this new product, and you wonder what chemist is able to prepare it.

We gave you in our communication of May 18 the exact contents of radioactive menthol iodine. We do not believe, however, that the preparation of this very delicate chemical composition may be confided to any chemist whatever, the same as one cannot have anti-diphtheritic serum, the anti-streptococcic serum, the vaccine virus of Jenner, or the serum against typhoid fever prepared by any chemist.

We are not at all desirous to keep our researches and results secret; we work honestly in broad daylight; we even proposed to the greatest hospitals of the world, and specially to several hospitals in England, to submit to our treatment a certain number of patients taken in the usual conditions, to prove in a striking manner the action and the efficiency of radioactive menthol iodine. Indeed, one should not submit to our treatment cachectic people or organisms labouring under generalised infection, for whom no serious attempt may be made with any hope of success.

We trust to your loyalty to publish the present letter, and remain, dear Sirs, with kindest regards,

Yours truly,
Dr. de Szendeffy, of Budapest.
Dr. L. Dieupart, of Paris.
Dr. S. Bernheim, of Paris.
A. Baud, Assistant de l'Euvre de la Tuberculose Humaine.

Paris, June 9, 1911.

[We are very glad to publish this letter, which is a fair reply to our criticism. We beg our colleagues to bear in mind, however, that our remarks were primarily directed against the senseless flaunting of medical canards in an ignorant lay Press. In justice to ourselves, too, we must state that we did not receive the communication referred to in the letter, and were therefore not in a position to judge with detailed knowledge of the composition of the new drug.—Ed. The Hospital.]

